# Analysis of Critical Dimensions for Nanowire Core-Multishell Heterostructures

**DOI:** 10.1186/s11671-015-1097-7

**Published:** 2015-10-06

**Authors:** Xin Yan, Shuyu Fan, Xia Zhang, Xiaomin Ren

**Affiliations:** State Key Laboratory of Information Photonics and Optical Communications, Beijing University of Posts and Telecommunications, Beijing, 100876 China

**Keywords:** Nanowire, Core-multishell, Quantum well, Critical dimension, 62.23.Hj, 68.65.Fg, 81.40.Jj

## Abstract

Critical dimensions for nanowire core-multishell heterostructures are analyzed by using finite-element method based on the energy equilibrium criteria. Results show that the nanowire core-shell heterostructure can sufficiently reduce the strain in the shell and increase the critical shell thickness. The critical dimensions for the nanowire core-multishell heterostructure are determined by the stress fields generated at two heterointerfaces. For thin barrier, the critical dimensions decrease as the core radius increases, while when the barrier is thick enough, the critical dimensions show an increase with the increase of core radius conversely. This can be attributed to a competition between the lattice mismatch and strain distribution, which dominate the critical dimensions alternatively. Two critical quantum well thicknesses are obtained in the nanowire core-multishell heterostructure. Below the dislocation-free critical thickness, the structure will be coherent regardless of the barrier thickness. While above the dislocation-unavoidable thickness, dislocations are always energetically favored. In the dislocation-controllable region between the two critical thicknesses, coherent structure can be obtained via controlling the well and barrier thicknesses. The results are in good agreement with the experimental data and may serve as guidance for the design of coherent nanowire core-multishell quantum well structures and devices.

## Background

In recent years, semiconductor nanowires (NWs) have attracted great attention due to their potential applications in electronic and photonic devices such as field effect transistors, lasers, photodetectors, and solar cells [1–4]. In comparison with homogeneous NWs, NW heterostructures can dramatically improve the performance and add advanced functionalities. For example, surface-passivated core-shell NW heterostructure can dramatically enhance the emission efficiency and electron mobility [5–7]. In comparison with homogeneous NWs, NW core-multishell quantum well (QW) heterostructures are more attractive in nanolasers due to the much stronger carrier confinement, decoupled cavity/gain-medium structure, as well as wavelength tenability [8–10]. However, due to the lattice mismatch between different materials, dislocations may generate in the NW heterostructures, which dramatically degrade the performance.

Theoretical analysis of the critical dimensions of NW heterostructures is helpful for designing advanced nanodevices with high crystal quality and feasible dimensions. Up to date, critical dimensions for axial NW heterostructures have been studied analytically and numerically [11–14]. In comparison with axial heterostructures, the strain release mechanism is quite different for core-shell heterostructures. For example, the lateral stress relaxation effect, which can sufficiently reduce the strain in NW axial heterostructures, is negligible in NW core-shell heterostructures due to a large interface area [11]. Instead, the nanosized curve surface of the NW could help to release a part of strain [15]. Although experiments on NW core-shell heterostructures have been widely reported [16–21], theoretical work on the critical dimensions of core-shell heterostructures is still limited [22–25]. Particularly, the study on critical dimensions for NW core-multishell heterostructures has not been reported yet, although which is significant for achieving high-performance optoelectronic devices. In this paper, we explore the coherency limits in NW core-shell and core-multishell heterostructures by using finite-element method (FEM) based on the energy equilibrium criteria. The effect of the core radius, barrier thickness, and lattice mismatch on the critical QW thickness is discussed and the design criteria for dislocation-free heterostructures are revealed. The theoretical results agree well with previously reported experimental values.

## Methods

**Fig. 1:**
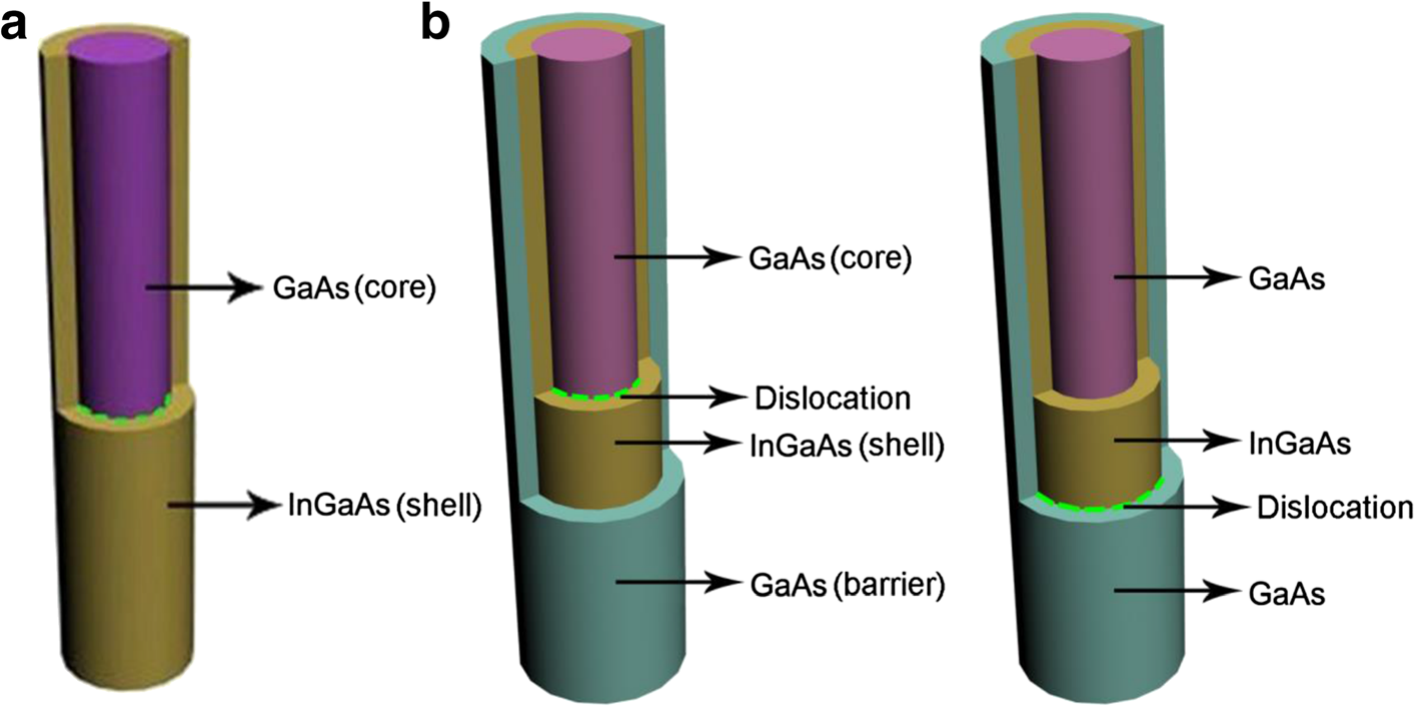
Schematic diagram of the NW core-shell (**a**) and core-multishell (**b**) heterostructures

The schematic diagrams of the NW core-shell and core-multishell heterostructures are shown in Fig. 1a and b respectively. Cylindrical coordinate system is used, in which the long axis of the NW corresponds to the zinc blende [111] direction. Simulations are designed for the specific GaAs/In_x_Ga_1-x_As material system and performed in the framework of linear-isotropic elasticity, where different Young’s moduli (*E*) and Poisson’s ratios (*v*) for different materials are taken into consideration.

When a core-shell heterostructure is formed between two different materials, the coherence requirement will result in tangential and longitudinal strain components due to the lattice mismatch [26]. As the radius of the wire is free to accommodate the strain, the stress normal to the interface is taken to zero. For isotropic materials, the strain component at the heterointerface is denoted by

1 $$ {\varepsilon}_{zz,\theta \theta }={f}_{zz,\theta \theta }=\frac{a_s - {a}_e}{a_e} $$

where *a*_*s*_ and *a*_*e*_ are the lattice parameters of the core and shell, respectively. The mismatch stress is generated and satisfies the equation as follows [27, 28]:

2 $$ {\sigma}_{ij}={c}_{ijkl}{\varepsilon}_{kl} $$

where *c*_*ijkl*_ is the elastic constant. The coherent elastic strain energy *W*_co_ is the integral of strain energy density *w* [29]:

3 $$ {W}_{co}={\displaystyle \underset{\varOmega }{\int }w\cdot dV}=\frac{1}{2}{\displaystyle \underset{0}{\overset{L}{\int }}dz}{\displaystyle \underset{0}{\overset{R}{\int }}dr}{\displaystyle \underset{0}{\overset{2\pi }{\int }}{\sigma}_{ij}{\varepsilon}_{ij}rd\theta } $$

where *Ω* is the volume of NW heterostructure, *L* is the length of NW, and *R* is the total radius of the structure. In this work, FEM is used to analyze the stress distribution and calculate the strain energy by dividing the whole system into small units and calculating the variables one by one.

To study the critical dimensions of the NW heterostructure, a loop dislocation is introduced at the heterointerfaces, as shown in Fig. 1 [30]. The dislocation allows partial relaxation of the lattice-mismatch-induced strain but also offers a strain field associated with itself. After the formation of the loop dislocation, a plane of atoms will be removed or inserted, and the strain constraint changes compared with the state of coherency. Thus, the mismatch strain expression can be written as follows [31]:

4 $$ {\varepsilon}_{zz}^{\hbox{'}}={f}_{zz}^{\hbox{'}}=\frac{a_s-{a}_e}{a_e}-nd $$

Here, the thermo-dynamic equilibrium approach is adopted. The energy associated with dislocations includes two parts: the loop dislocation-induced energy (*W*_loop_) and the residual strain energy (*W*_res_). *W*_res_ can be obtained by Eq. (3), while *W*_loop_ can be obtained from the following equation [28]:

5 $$ {W}_{\mathrm{loop}}=\frac{R\mu {b}^2}{2\left(1-\nu \right)}\left( \ln \frac{32R}{b}-1\right) $$

Where *R* is the radius of the dislocation loop, *μ* is the shear modulus, and *b* is Burgers vector. According to the energy equilibrium criteria, the structure prefers to stay at the lowest energy state. Thus, the finial energy state can be determined by:

6 $$ \varDelta W={W}_{\mathrm{dis}}-{W}_{\mathrm{co}}={W}_{\mathrm{res}}+{W}_{\mathrm{loop}}-{W}_{\mathrm{co}} $$

Where *W*_dis_ is the total energy with a loop dislocation, *W*_co_ is the coherent elastic strain energy, *W*_res_ is the residual strain after introducing a dislocation, and *W*_loop_ is the loop dislocation-induced energy. If ∆*W* > 0, the NW tends to be coherent. Whereas if ∆*W <* 0, dislocations will generate.

## Results and Discussion

### Critical Dimensions for the NW Core-Shell Heterostructure

**Fig. 2:**
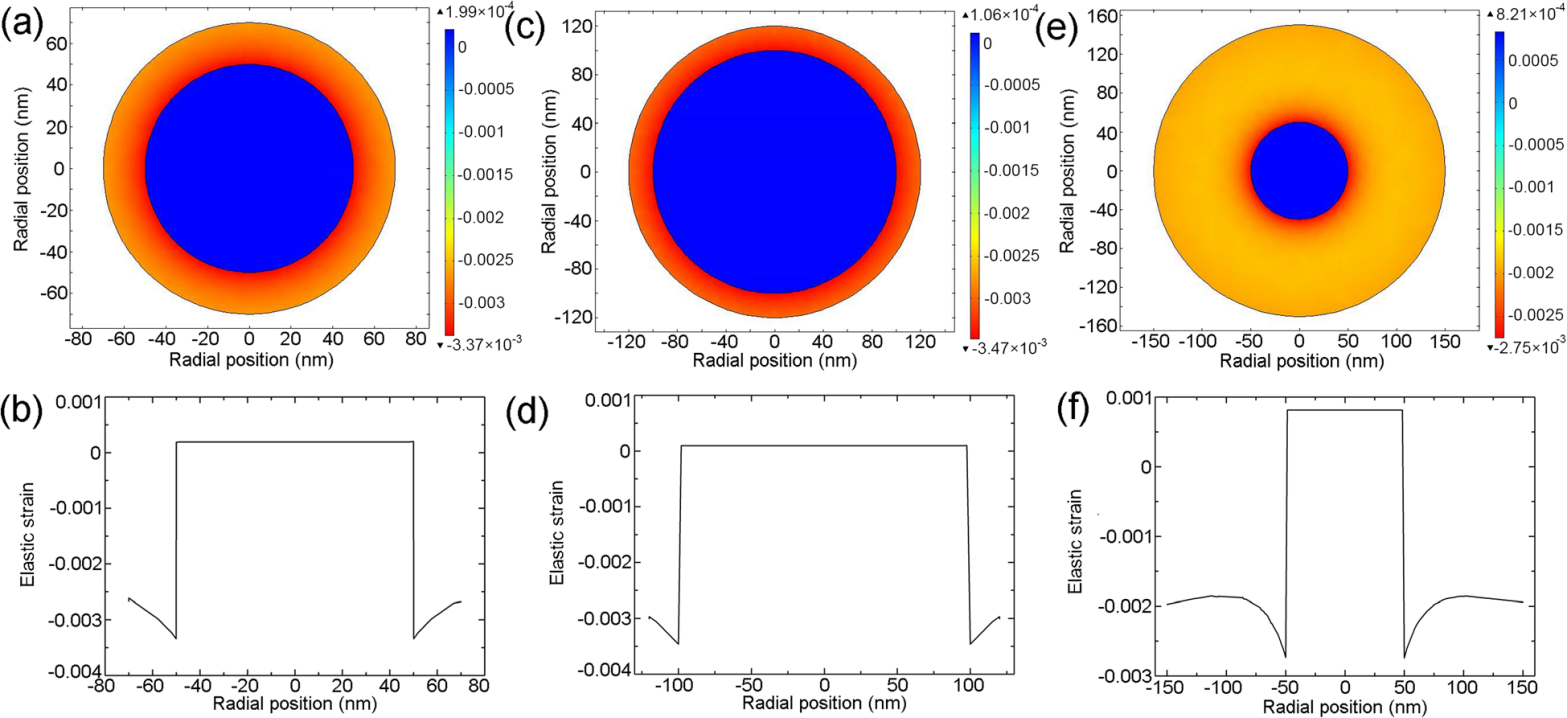
Stress field distribution along the radial direction of a NW core-shell heterostructure. The core radius and shell thicknesses are (50, 20), (100, 20), and (50, 100) nm in (**a**, **b**), (**c**, **d**), and (**e**, **f**), respectively

Figure 2 shows the distribution of stress field over the cross section of three GaAs/In_0.2_Ga_0.8_As NW core-shell heterostructures with a core radius and shell thickness of 50, 20 nm (a, b), 100, 20 nm (c, d), and 50, 100 nm (e, f), respectively. From Fig. 2a, c, e, we can see that stress fields are generated in both the core and shell, and the core suffers a tensile strain while the shell suffers a compressive strain. This suggests the ability of NW core-shell heterostructure to distribute strain between the core and shell. For a thin shell of 20 nm, the strain energy is mainly concentrated in the shell, as shown in Fig. 2b, d. This is similar to the thin film epitaxy [32]. Comparing Fig. 2b, d, we can see that for a certain shell thickness, the strain in the shell increases while in the core decreases, as the core radius increases. As the shell thickness increases to 100 nm, a substantial portion of strain is passed to the core, resulting in a dramatic decrease of strain in the shell, as shown in Fig. 2f. From Fig. 2b, d, f, we can also see that the strain remains nearly constant in the core region, while the strain in the shell decreases from the core/shell interface to the shell surface. This can be explained by a specific strain self-releasing effect in NW core-shell heterostructure. Due to the nanosized curve surface of the NW, the shell can release the strain induced by the lattice mismatch between the core and shell itself [15, 33, 34]. Thus, the strain becomes weaker and weaker as the shell thickness increases.

**Fig. 3:**
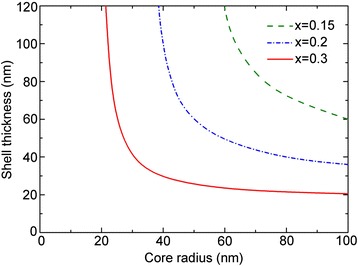
Dependence of the critical shell thickness on the core radius for different lattice mismatch in a GaAs/In_x_Ga_1-x_As system

In a planar lattice-mismatch system, as the epitaxial film thickness increases, the strain energy increases and dislocations will appear. The critical thickness can be defined as the film thickness at which the film is no longer coherent with the substrate due to strain relaxation via the formation of dislocations [22]. In contrast to planar films, for the NW core-shell heterostructure, due to the comparable volumes of the core and shell regions, both the core and shell should be considered. For a certain core radius, there exists a critical shell thickness and vice versa. The critical dimensions of a NW core-shell system are combinations of core and shell dimensions that will lead to coherently strained structures. The dependence of the critical shell thickness as a function of the core radius for different lattice mismatch is shown in Fig. 3. Under a certain mismatch, there is a critical core radius, below which the NW heterostructure will be coherent regardless of shell thickness. For NW core-shell heterostructure with a core radius larger than the critical core radius, there is a critical shell thickness, below which no dislocations will occur. For a GaAs/In_0.2_Ga_0.8_As NW core-shell heterostructure with a core radius of 100 nm, the critical In_0.2_Ga_0.8_As shell thickness is determined to be 37 nm, which is much larger than that of In_0.2_Ga_0.8_As film grown on planar GaAs substrate (about 14 nm calculated by this model). This suggests that the NW core-shell structure can sufficiently reduce the strain in the shell via passing the strain into the core as well as the strain self-releasing effect.

### Critical Dimensions for the NW Core-Multishell Heterostructure

**Fig. 4:**
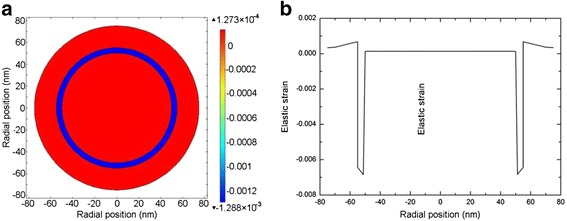
Stress field distribution along the radial direction of a NW core-multishell heterostructure. The core radius, well thickness, and barrier thickness are 50, 5, and 20 nm, respectively

The stress distribution over the cross section of a GaAs/In_0.2_Ga_0.8_As/GaAs NW core-multishell heterostructure is shown in Fig. 4a, b. In the reported experiments, the NW core-multishell QW heterostructure typically has a thin QW of several nanometers for a strong quantum confinement as well as obvious quantum size effect and thick barriers, i. e. core and shell, of several dozen to hundred nanometers for a sufficient confinement of carriers [10, 35–37]. However, if the core and shell are too thick, the effect of such a thin QW is negligible. Thus in the simulation, the core radius, QW thickness, and barrier thickness are set to be 50, 5, and 30 nm, respectively, for a better presentation of the stress distribution. We can see that the strain field is generated in all the three parts, suggesting that both the core and barrier share the strain via elastic deformation. The core suffers a constant tensile strain as observed in the core-shell structure. The well and barrier suffer a compressive and tensile strain, respectively, and both the strain decrease from the inner to the outer, which are indicatives of the strain self-releasing effect.

**Fig. 5:**
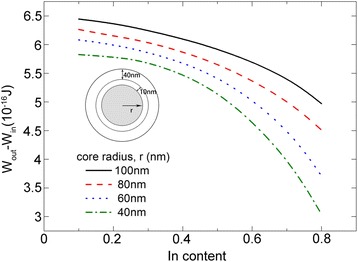
Dependence of the total energy difference with dislocations generated at the inner and outer interface on In content and core radius

As shown in Fig. 1b, dislocations may generate at either the inner heterointerface between the core and QW or the outer heterointerface between the QW and the barrier, depending on the total energy. Figure 5 shows the dependence of the energy difference (∆*W*, defined as the difference between the total energy with a dislocation generated at the outer heterointerface and at the inner heterointerface) on the In content and core radius. We can see that ∆*W* decreases with the increase of In content and decrease of the core radius. Nevertheless, ∆*W* is always positive, suggesting that the dislocation prefers to generate at the inner heterointerface due to a lower energy.

**Fig. 6:**
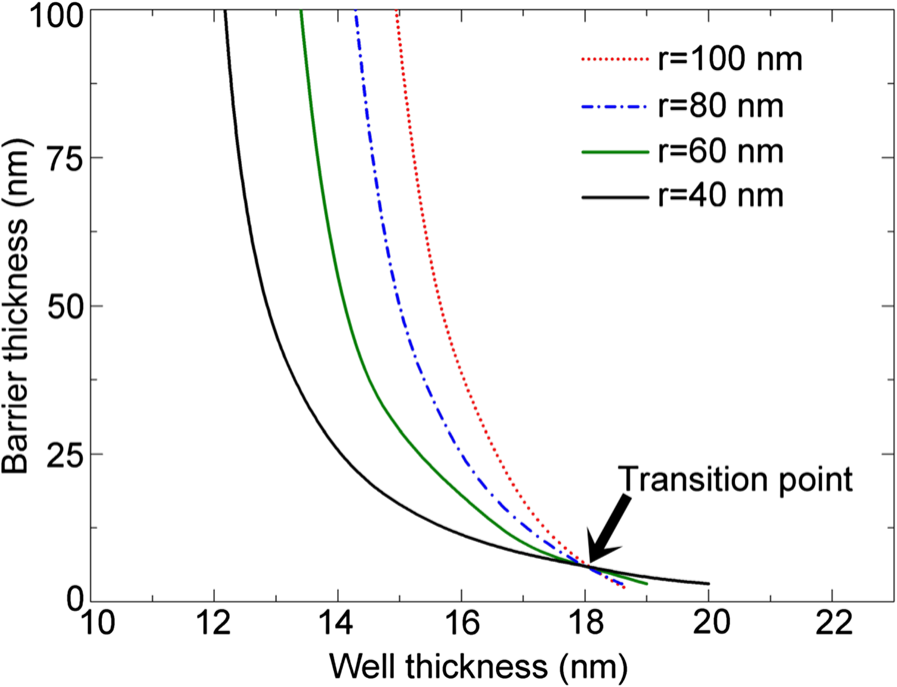
Critical dimensions of NW core-multishell heterostructures for different core radius. The In content in the QW is 0.3

Now, we turn to study the critical dimensions for the NW core-multishell QW heterostructures. Figure 6 shows the dependence of the critical barrier thickness on the QW thickness for different core radius. The In content in the In_x_Ga_1-x_As QW is 0.2. We can see that the critical barrier thickness decreases as the QW thickness increases. For a certain core radius, there exists a critical QW thickness, below which the NW core-multishell QW heterostructure will be coherent regardless of the barrier thickness. For a NW core-multishell QW heterostructure with a QW thickness larger than the critical QW thickness, there is a critical barrier thickness, below which no dislocations will occur. In addition, the core radius has a strong influence on the critical dimensions of the structure, characterized by a shift of the critical curve as the core radius varies. The curve group for different core radii can be divided into two regions, with a turning QW thickness of 18.1 nm. When the QW thickness is less than 18.1 nm, the curve moves left as the core radius increases, suggesting a reduction of the critical barrier thickness and QW thickness. Once the QW thickness exceeds 18.1 nm, the curve exhibits an opposite right shift as the core radius increases. This can be explained by a competition between the lattice mismatch and strain distribution. For a NW heterostructure, the lattice mismatch dominates the total strain energy, while the strain distribution determines the strain stored in each part. The critical dimensions of NW heterostructures are jointly determined by the two factors. For a NW core-shell heterostructure with a certain shell thickness, as the core radius decreases, the strain in the shell is reduced as more strain energy is passed into the core, as shown in Fig. 2. Thus, the shell is more GaAs-like for a smaller core radius and more InGaAs-like for a larger core radius. When the GaAs barrier is grown on the InGaAs shell (QW), the lattice mismatch between the QW and barrier is larger for a smaller core radius, resulting in a smaller critical barrier thickness. This can explain the left shift of the critical curve, suggesting that the lattice mismatch dominates the critical dimensions for smaller QW thickness. On the other hand, as the core radius decreases, more strain is passed to the core and QW, and the strain in the barrier is reduced. This can lead to a right shift of the critical curve, suggesting that the strain distribution dominates the critical dimensions for larger QW thickness. We also find a phenomenon that as the core radius increases, the shift of curve becomes slower. This is because that as the core radius increases, the NW structure is more like a planar epitaxial heterostructure. Most of the strain is stored in the QW and barrier, and the lattice mismatch between the QW and barrier, as well as the strain distribution among the three parts, changes little with the core radius.

**Fig. 7:**
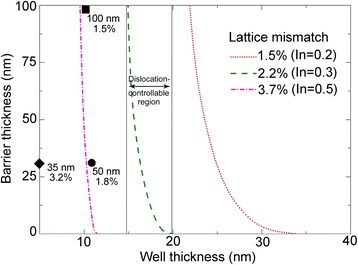
Critical dimensions of NW core-multishell heterostructures for different lattice mismatches. *Symbols* give the experimental values of NW core-multishell heterostructures without dislocations for certain lattice mismatch and core radius. The *circle*, *square*, and *diamond* represent GaAs/In_0.25_Ga_0.75_As [10], GaAs/In_0.2_Ga_0.8_As [35], and InP/InAs material systems [36], respectively

The critical dimensions of the NW core-multishell heterostructure with a core radius of 100 nm for different lattice mismatch are shown in Fig. 7. Unlike the critical dimensions of the NW core-shell structure, there exist two critical QW thicknesses. We take the GaAs/In_0.3_Ga_0.7_As/GaAs system for example. The dislocation-free critical QW thickness is about 15 nm, and below this thickness, the structure will be coherent regardless of the barrier thickness. The dislocation-unavoidable critical QW thickness equals to the critical shell thickness of a NW core-shell heterostructure (about 20 nm), above which dislocations are always energetically favored. Between the two critical thicknesses is the dislocation-controllable region. In this region, coherent structure can be obtained via controlling the dimensions of the QW and barrier thickness. Symbols in the image show some experimental data of NW core-multishell QW heterostructures without dislocations. Square, circle, and diamond represent the system with lattice mismatch of 1.5, 1.8, and 3.2, respectively [10, 35, 36]. It can be seen that the simulated results are in good agreement with the experimental data. For example, for the GaAs/In_0.2_Ga_0.8_As/GaAs NW core-multishell QW heterostructure (1.5 % lattice mismatch) with a core radius of 100 nm and barrier thickness of 100 nm, the dislocation-free critical QW thickness is calculated to be 22 nm. In the experiment, the QW thickness is about 10 nm and the system is dislocation-free, in good agreement with the theoretical results [10]. The results also show that the critical well thickness in a NW core-multishell QW heterostructure is slightly thicker than that in a planar QW counterpart (for GaAs/In_0.2_Ga_0.8_As/GaAs system, the critical well thickness in a NW heterostructure is about 22 nm, while in a planar heterostructure is 20 nm) [38] and can be much larger via barrier engineering.

## Conclusions

In summary, critical dimensions for NW core-shell and core-multishell heterostructures are analyzed theoretically. Results show that the NW core-shell structure can sufficiently reduce the strain in the shell and increase the critical shell thickness via passing the strain into the core as well as a self-releasing mechanism. After introducing a barrier to form a NW core-multishell QW heterostructure, the critical QW thickness decreases compared with a core-shell structure, suggesting an increased strain due to the lattice mismatch between the well and barrier. The lattice mismatch and strain distribution dominate the critical dimensions alternatively, resulting in a decrease of critical dimensions for thin barrier and an increase for thick barrier, with the increase of core radius. Two critical QW thicknesses are obtained, which give the criteria of coherent NW core-multishell QW structures. The results may serve as guidance for the design of radial NW heterostructures and devices.

## Abbreviations

FEM: Finite-element method
NW: Nanowire
QW: Quantum well
